# miR-154 inhibits migration and invasion of human non-small cell lung cancer by targeting ZEB2

**DOI:** 10.3892/ol.2021.12873

**Published:** 2021-06-18

**Authors:** Xingyu Lin, Zhiguang Yang, Peng Zhang, Yunpeng Liu, Guoguang Shao

Oncol Lett 12: 301-306, 2016; DOI: 10.3892/ol.2016.4577

Following the publication of this paper, it was drawn to the Editors' attention by a concerned reader that certain of the western blotting data shown in Figs. 3B, 4B and 5B bore unexpected similarities to data appearing in different form in other articles by different authors. Owing to the fact that some of the contentious data in the above article had already been published elsewhere, or were already under consideration for publication, prior to its submission to *Oncology Letters*, the Editor has decided that this paper should be retracted from the Journal. After having been in contact with the authors, they agreed with the decision to retract the paper. The Editor apologizes to the readership for any inconvenience caused.

